# An improved and convenient petri plate-based method for studying the root growth of plants

**DOI:** 10.1016/j.mex.2023.102505

**Published:** 2023-11-30

**Authors:** Anish Kaachra, Anantika Suri, Sanjay Kumar

**Affiliations:** a Biotechnology Division, CSIR-Institute of Himalayan Bioresource Technology, Palampur, Himachal Pradesh 176061, India; b Academy of Scientific and Innovative Research (AcSIR), Ghaziabad, Uttar Pradesh, India; c Department of Biotechnology, Guru Nanak Dev University, Amritsar, Punjab 143005, India; d Agricultural Scientists Recruitment Board, Krishi Anusandhan Bhavan-1, Pusa, New Delhi 110012, India

**Keywords:** Root growth, Plate system, Vertical growth, Nutrient media, An improved and convenient petri plate-based method for studying the root growth of plants

## Abstract

Plant scientists across the globe are interested in studying the root growth architecture of plants to understand different processes determining growth and development in plants. In the majority of cases, root growth-related experiments are carried out on petri plates filled with solid nutrient media. However, plants growing in these plates are often exposed to conditions that are not close to the natural conditions. Also, it is difficult to pour two different media on the same plate which is quite a useful feature to study the effect of specific treatment on plant growth. In the present work, we describe an improved and easy-to-use petri plate system useful for studying root growth characteristics of young plants grown over solid nutrient media. In comparison to the conventional methods, the present plate system offers an advantage in terms of facilitating the pouring of two different media in the same plate, avoiding contact of the aerial part of the plant with nutrient media, and ensuring the growth of roots under dark conditions. The described plate, therefore, provides a convenient system to study root growth under conditions close to natural conditions and hence minimizing experimental artifacts.

• The plate system facilitates the pouring of two different nutrient media into the same plate.
• The aerial part of the seedling does not come in contact with the media.
• Ensures growth of roots under dark conditions.

## Graphical abstract

**Figure:**
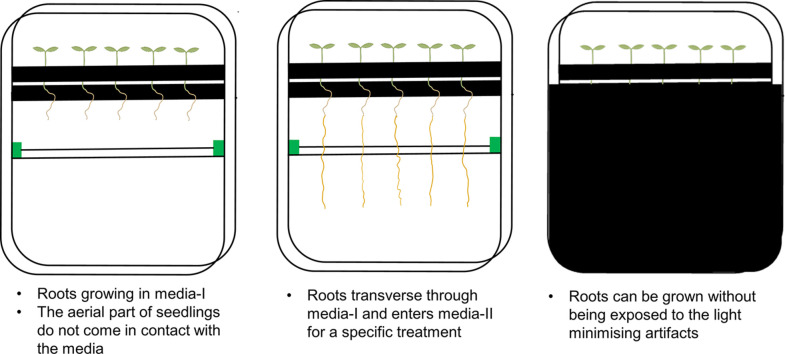

Specifications table

**Table:** 

Subject area:	Agricultural and Biological Sciences
More specific subject area:	Plant physiology
Name of your method:	An improved and convenient petri plate-based method for studying the root growth of plants
Name and reference of original method:	Not applicable
Resource availability:	All the information/resources related to the present method is available with corresponding author.

## Introduction

Root system architecture represents spatial arrangement of roots that play an important role in the growth and survival of plants. The root system exhibits broad plasticity characterized by shape, thickness, distribution, branching, length, and number of both primary and secondary roots [1,2]. Plant biologists are primarily interested in studying root architecture to understand the key genetic and environmental factors affecting root system architecture and hence plant growth [3,4]. Plate experiments for root growth analyses are essential for studying the effect of different inducers or inhibitors on plant growth and also to evaluate the performance of plants engineered for improved stress tolerance, higher nutritional uptake, etc. In general, root growth experiments are carried out using clear circular or square petri plates placed vertically in controlled environmental conditions. These plates are prepared by adding nutrient media supplemented with agar as a solidifying agent. A portion of solidified media has to be removed from the plate before placing the seeds or seedlings for root growth analysis. However, this method not only wastes the poured media but also increases the chance of contamination. Another method is growing the aerial part of seedlings outside the plate, however, this method exposes nutrient media to the outside environment which increases the chances of contamination. Further, it is difficult to pour two different nutrient media in the same plate.

In some experiments, seedlings must first be established in the standard media before being transferred to media with a different composition or with the addition of an inducer or inhibitor of choice. The process of transferring the seedlings could injure the roots and raise the possibility of contamination. We in the present study describe a plate system that circumvents these problems by providing two sections across the length of the plate. Each section is provided with an aperture that can be covered with sterile tape. The design of this plate makes it easier to pour two different media into the same plate without the need of removing solidified media.

Another limitation of using regular petri plates is that after a certain period of growth, the aerial part of the plant comes in contact with the nutrient media which may affect root system architecture. For example, a study showed that direct contact of aerial tissue with sucrose in the nutrient media promoted lateral root formation in *A. thaliana* [5]. In yet another study, it was found that shoot-supplied ammonium in agar plates inhibited lateral root formation in *A. thaliana* [6]. There are studies where the aerial part of a seedling has been grown outside the plate through holes made in media-filled plates [[7], [8], [9]]. However, it is difficult to make holes at uniform positions particularly when the experiment includes a series of treatments and/or replicates. In addition, holes in the plate may become entry points for different contaminants ruining the experiment. The plate described in the present study has a partition in the upper section which provides a barrier between the aerial part and the nutrient media. This construction helps in avoiding any artifacts resulting from contact of the aerial part of the plant with the nutrient media.

Under natural conditions, plant roots grow in complete darkness while only shoots are illuminated throughout their life cycle. The light has a pronounced effect on the root physiology and therefore artifacts may result from growing plants in transparent petri plates [[10], [11], [12]]. In a previous study, an improved agar plate method was described wherein seedlings were grown outside the black plates through holes provided at the top cover of the plate [9]. However, as discussed earlier, holes in the plate may cause contamination. In yet another study, Silva-Navas et al. [13] devised a Dark-Root (D-Root) system to avoid exposure of the roots to the light. In this system, a black methacrylate comb was fitted inside the solidified media and the square petri plate was placed inside a black methacrylate box, which allowed roots to grow in the dark while the aerial portions were illuminated. Our plate system provides a simpler solution to avoid exposure of roots to the light by providing a black partition in the upper section which blocks light exposure to the roots. After the transfer of seedlings, the remaining openings in the upper section can be covered with black sterile tape to further block any light exposure to roots. Also, a self-adhesive black film is provided to cover the root-growing area of the base plate, ensuring the growth of roots under dark conditions.

The following sections provide details on the construction and method to use the plate described in the present study.

## Method details

### Construction of plate for root growth analysis

**Fig. 1:**
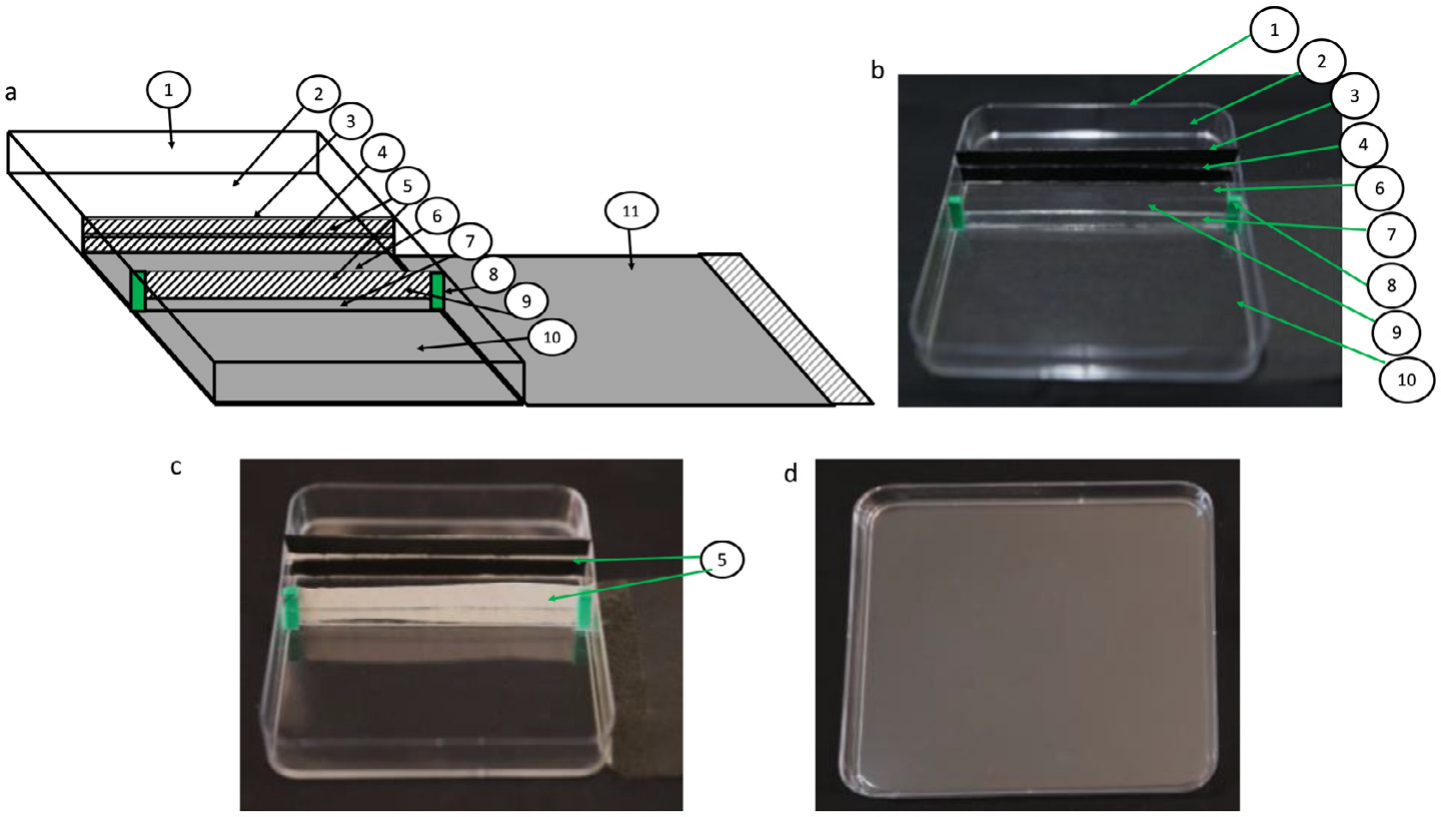
Plate construction for root growth analysis. (a) schematic diagram and (b) base plate with different components labelled as 1, base petri plate; 2, empty section for growth of aerial part of plants; 3, upper black partition; 4, narrow aperture in the upper partition for holding seeds or seedlings; 5, sterile tape to cover apertures in the upper and lower partitions of the plate; 6, upper section for pouring nutrient Media-I; 7, lower partition; 8; hinges on the sides of lower partition to hold sterile tape; 9, wider aperture in lower partition for growth of roots in Media-II; 10, lower section for pouring nutrient Media-II, 11, black film to cover the root growing region of the plate (c) apertures in the upper and lower partition of the plate sealed with sterile tape, and (d) cover plate. The upper partition has a narrow aperture so that the aerial part does not come in contact with the nutrient media while the wider aperture in the lower partition facilitates the entry of roots in the Media-II without obstruction.

An optically clear, transparent square plate of (120 mm × 120 mm × 12 mm) size was used as a base plate. At a distance of 40 mm from one side of the plate, two pieces of black glass, each of 118 mm × 5 mm size, are fixed parallel to each other leaving a gap of 2 mm. This resulted in the upper black partition with a narrow horizontal aperture. For a lower partition, a single piece of glass (118 mm × 5 mm) is fixed below the upper partition. Two small hinges are provided at each side of the lower partition to support the sterile tape and to create a wider aperture in the lower section (Supplementary Fig. S1). The base plate along with different components are shown in Fig. 1a–d. A sterile tape is used to seal the apertures in the upper and lower partitions of the plate before media pouring. A self-adhesive black-colored film is used to cover the root-growing region of the plate.

### Plant material and growth conditions

Seeds of *Brassica juncea* were surface sterilized using the method described previously [14]. For root growth, Murashige and Skoog media (MS media; [15]) without nitrogen (N) source containing 2 % sucrose and 0.8 % agar was used as a basal media. Media-I consisted of 10 mM NH_4_NO_3_ and 10 mM KNO_3_, whereas Media-II consisted of 1 mM NH_4_NO_3_ and 1 mM KNO_3_ as a nitrogen (N) source. Seedlings were grown over plates at 25 °C and a 16 h light/8 h dark photoperiod (photosynthetic photon flux density, 150 µmol m^−2^ s^−1^) and 70 % relative humidity.

### Step-wise method for preparation of plate for root growth analysis

**Fig. 2:**
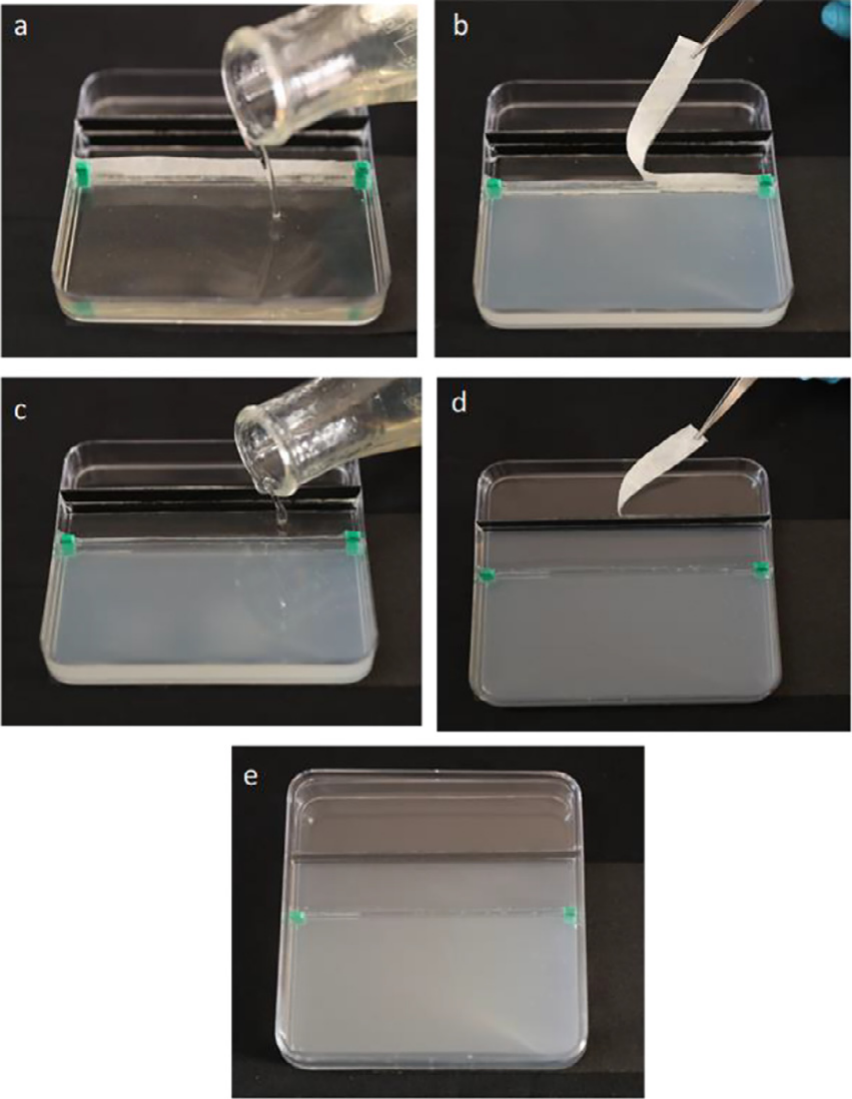
Step-wise method for preparing the plate system for root growth experiments (a) Sterile Media-II was poured in the lower section of the plate and allowed to solidify, (b) After solidification of Media-II, sterile tape on the lower partition was removed, (c) Sterile media-I was poured in the upper section of the plate and allowed to solidify, (d) After solidification, sterile tape on the upper black partition was removed, (e) Base plate was closed with cover plate and ready for transfer of seeds for root growth analysis.
(IV) After solidification of the Media-II, remove the sterile tape on the lower partition using sterilized forceps (Fig. 2b).
(V) Pour sterile Media-I into the upper section between the upper and lower partitions of the plate and allow it to solidify (Fig. 2c).
(VI) After solidification of the Media-I, remove the sterile tape on the upper partition (Fig. 2d).
(VII) Place the sterilized seeds of B. juncea over the aperture provided in the upper partition of the plate.
(VIII) After the transfer of seeds close the plate with a cover plate (Fig. 2e) and seal using sterile surgical tape. The root-growing region of the plate was covered with black film to grow roots under dark conditions.
(IX) Twelve-days old seedlings of B. juncea growing over plates poured with nutrients Media-I and Media-II are shown in Fig. 3a &b.

The following sequence should be followed for the preparation of the plate for root growth analysis.

(I) Autoclave the two different nutrient media (Media-I and Media-II).
(II) Before media pouring, both media should be cooled at room temperature for some time without allowing solidification and apertures on the upper and lower sections should be sealed with sterile tape.
(III) In a laminar air flow hood, pour sterile Media-II into the lower section of the plate and allow it to solidify (Fig. 2a).

**Fig. 3:**
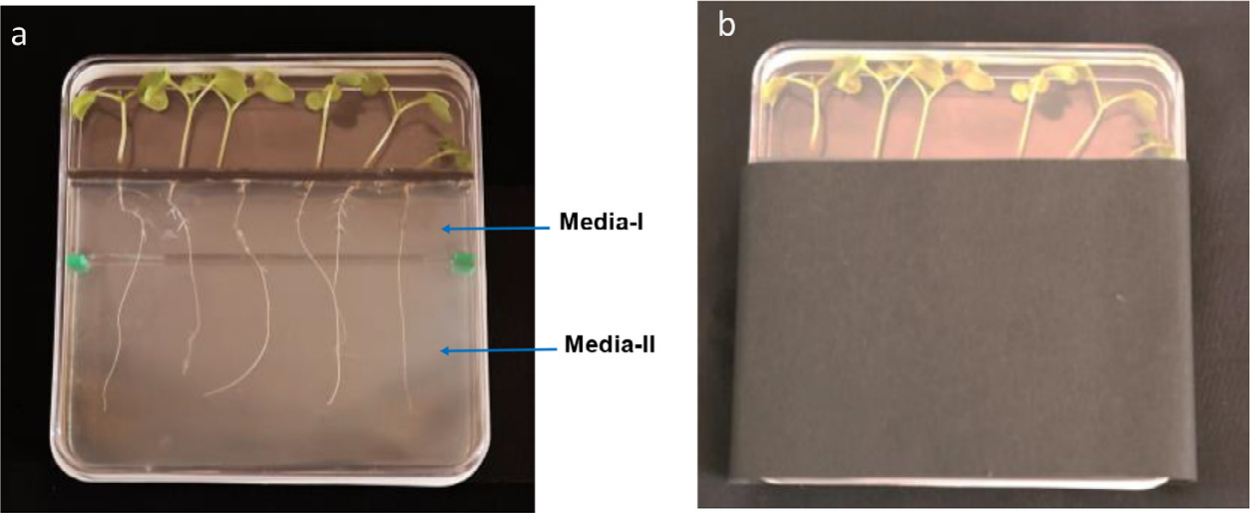
Photographs showing (a) Twelve-days old seedlings of *B. juncea* growing over plates containing media-1 and media-II as described in the methods section (b) plates showing seedlings of *B. juncea* growing over plates with root growing region covered with black film.

## Conclusions

It is noteworthy that studies on root system architecture yield valuable information on different molecular and physiological aspects of plant growth and development. Under laboratory conditions, majority of these experiments are carried out using clear circular or square petri plates filled with solid nutrient agar media. However, the use of these plates is often associated with certain limitations which may lead to experimental artifacts. For instance, with conventional plates, it is difficult to pour two different media into the same plate. This is particularly important for experiments aiming to study the effect of different treatments on root growth. In such studies, it is important that seedlings first get established on the normal optimum media as direct exposure to treatment may inhibit the growth of seedlings. Notably, transfer of seedlings from plates with normal media to the plates with media for a specific treatment may cause root damage. The plate described in the present work facilitates the pouring of normal media in the upper section of the plate and media for a specific treatment in the lower section. This design would let seedlings grow first on normal media, and then after some time, the roots would transverse from normal media to the media for a particular treatment as illustrated in Fig. 3a. This would facilitate giving any treatment to the roots and a range of experiments can be executed. Further, there is no need for cutting and removing the media from the plate which may lead to contamination.

Previous studies have shown that direct contact of the aerial part of seedling with the nutrient media may cause artifacts in root growth ([[5], [6]]; Dubrovsky et al. [16]). Further, without any physical separation, it is difficult to prevent this contact. As a solution to this limitation, there are studies where the aerial part of the shoot was grown outside the plate through holes made on one side of the plate [9,17]. While making these holes for multiple plates is a difficult task, these holes could also become the entry point for different contaminants. The plate described in the present study circumvents this problem by providing an upper partition with a narrow aperture which besides supporting seeds or seedlings, provides a physical separation of the aerial part of the seedling and the nutrient media. Further, the present plate system ensures the growth of the aerial part of the seedling in the light whereas the root grows in the dark. This feature is much needed to keep the experimental conditions close to the natural conditions as realized in the previous studies also [9,17]. Notably, a d-Root system has been described previously which allowed the growth of roots under dark while only the aerial part of the shoots was illuminated [13]. This system required petri plate to be placed inside the black methacrylate box and also involved insertion of a black methacrylate comb into the solidified media. Nevertheless, the d-Root does not specify any measures to avoid contact of the seedling's aerial portion with the nutrient medium, nor does it permit the pouring of two different media in a single plate. Our plate system provides a simpler construction to avoid the exposure of the roots to the light. For this, a black upper partition is provided and the root-growing region of the plate is covered with self-adhesive black film (Fig. 3b). The space between the seedlings on the upper section can be sealed with black sterile tape to ensure the growth of roots under complete darkness.

Collectively, we describe a plate system that offers many advantages as compared to the conventional plate for root growth analysis (Supplementary Table S1). We believe that the present plate system would be of great interest to plant scientists, and young researchers who are growing plants for root growth analysis under sterile conditions.

## Ethics statements

This work does not involve living animals, and no consent is needed. Ethical approval is not applicable.

## Funding

The present work was supported by the 10.13039/501100001412Council of Scientific and Industrial Research (CSIR), India, under Grant MLP201.

## CRediT authorship contribution statement

**Anish Kaachra:** Conceptualization, Methodology, Validation, Resources, Writing – original draft. **Anantika Suri:** Methodology, Formal analysis, Resources, Writing – review & editing. **Sanjay Kumar:** Project administration, Supervision.

## Declaration of Competing Interest

The authors declare that they have no known competing financial interests or personal relationships that could have appeared to influence the work reported in this paper.

## Data Availability

No data was used for the research described in the article.

## Appendix Supplementary materials

Image, application 1
