# Supplementary material for: An improved and convenient petri plate-based method for studying the root growth of plants
Source: MethodsX. 2023 Nov 30;12:102505. doi: 10.1016/j.mex.2023.102505 (PMC10755038; doi:10.1016/j.mex.2023.102505)
Supplement: Supplementary file 1 [file mmc1.docx]

**Supplementary file** **caption: Details on the plate system convenient for root growth analysis**


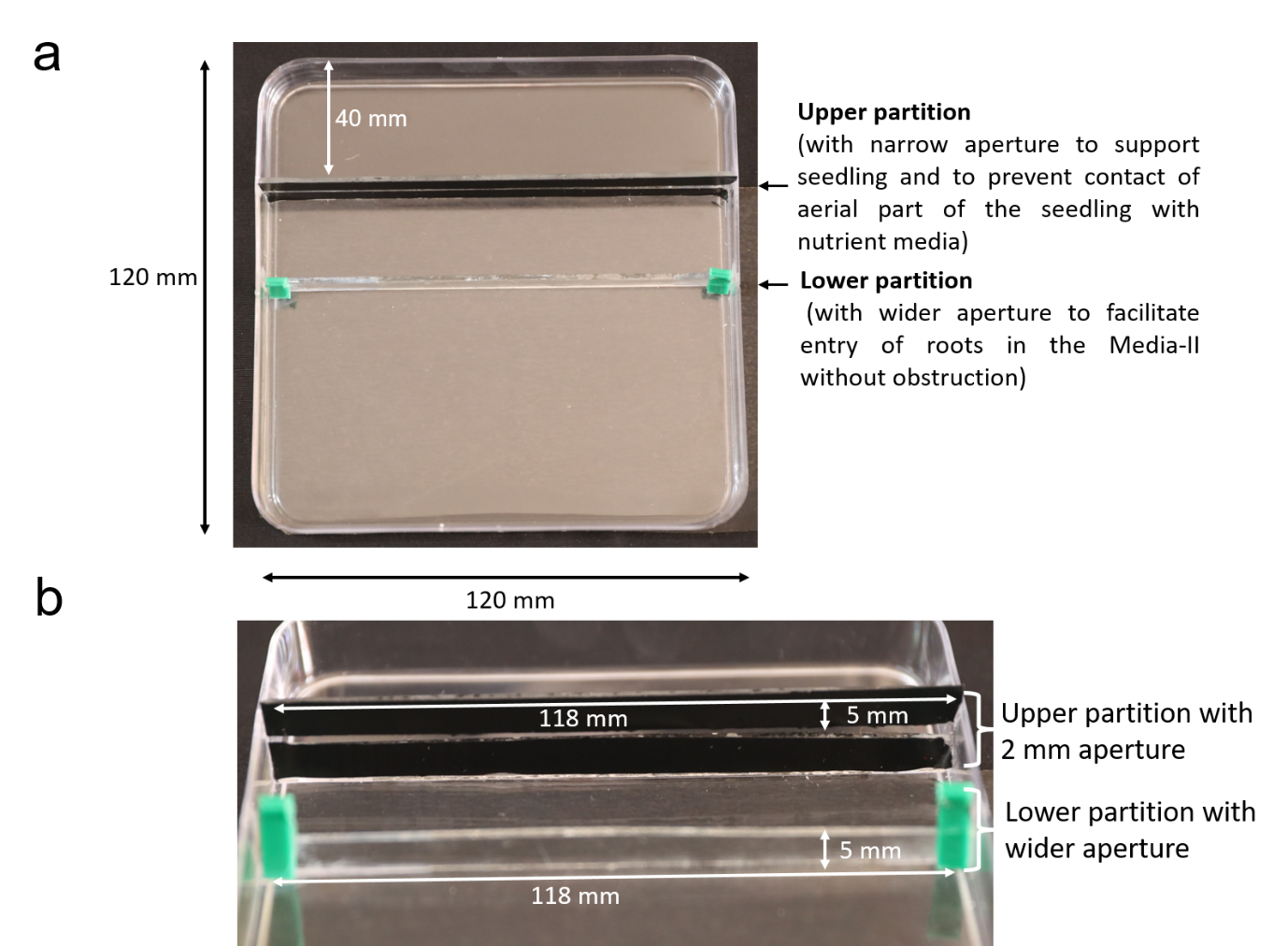


**Supplementary Fig. S1 (a)** Top view of the base plate with respective dimensions and position of upper and lower partition. **(b)** Magnified image showing upper and lower partitions with respective dimensions and apertures as mentioned in the Method details.

**Supplementary Table S1:** Key features distinguishing the present plate system from the conventional petri plates used for root growth analysis

| Sr. no. | Feature | Conventional petri plate | Present plate system |
| --- | --- | --- | --- |
| 1. | Provision of pouring two different media in the same plate | No | Yes |
| 2. | Physical separation of aerial part of the seedling from the nutrient media without making any holes in the plates | No | Yes |
| 3. | Removal of solidified media before seed transfer | Yes*  * Studies where solidified media has not been removed, there is a need to make holes in plate to grow seedlings which increases the chance of contamination. Also, when the seedlings were grown inside the plate, the aerial part of seedling comes in contact with the media resulting in experimental artifacts. | No |
| 4. | Provision of root growth under dark conditions | Yes*  * To ensure the root growth under dark conditions, the whole plate was covered with black film and seedling were grown outside the plate (Xu et al., 2013) through holes which may cause contamination. In other studies, additional materials were required to block light exposure to the roots. e.g., use of methacrylate box and methacrylate comb (Silva-Navas et al., 2015). | Yes  (without the need of making holes or use of any other additional materials) |

References:

- J. Silva‐Navas, M.A. Moreno‐Risueno, C. Manzano, M. Pallero‐Baena, S. Navarro‐Neila, B. Téllez‐Robledo, J.M. Garcia‐Mina, R. Baigorri, F.J. Gallego, J.C. del Pozo, D‐Root: a system for cultivating plants with the roots in darkness or under different light conditions, The Plant J. 84 (2015) 244-255. doi: [10.1111/tpj.12998](https://doi.org/10.1111/tpj.12998)
- W. Xu, G. Ding, K. Yokawa, F. Baluška, Q.F. Li, Y. Liu, W. Shi, J. Liang, J. Zhang, An improved agar-plate method for studying root growth and response of *Arabidopsis thaliana*. Sci. Rep. 3 (2013) 1273. doi: 10.1038/srep01273
